# In vitro digestive system simulation and anticancer activity of soymilk fermented by probiotics and synbiotics immobilised on agro-industrial residues

**DOI:** 10.1038/s41598-024-68086-3

**Published:** 2024-08-09

**Authors:** Abdallah I. Gad, Mona M. Orabi, Khadiga A. Abou-Taleb, Dina Y. Abdelghani, Shimaa A. Amin

**Affiliations:** 1 Agricultural Microbiology Department, Faculty of Agriculture, Ain Shams University, Hadayek Shobra, P.O. Box 68, Cairo, 11241 Egypt; 2 Department of Special Food and Nutrition, Agriculture Research Center, Food Technology Research Institute, Giza, Egypt

**Keywords:** Soymilk, Probiotic bacteria and synbiotic, Agro-industrial wastes, Encapsulation, Freeze-dried, Gastrointestinal track, Cytotoxicity, And anticancer, Biotechnology, Microbiology

## Abstract

In this study, a variety of probiotic strains, including *Lactiplantibacillus plantarum, Lacticaseibacillus casei, Lactobacillus acidophilus, Streptococcus thermophilus, Bifidobacterium longum, Limosilactobacillus reuteri, Lactobacillus delbrueckii* subsp. *bulgaricus, Lacticaseibacillus rhamnosus,* and *Bifidobacterium bifidum*, were utilized for soymilk fermentation both as free cells and as synbiotics on agro-industrial residuals such as okara, whey protein, banana peels, apple pomace, sugarcane bagasse, orange peels, and lemon peels. Among these, *Lacticaseibacillus rhamnosus* emerged as the most significant strain for soymilk fermentation, exhibiting a viability of 10.47 log cfu/mL, a pH of 4.41, total acidity of 1.12%, and organic acid contents (lactic and acetic acid) of 11.20 and 7.50 g/L, respectively. As a synbiotic *Lacticaseibacillus rhamnosus* immobilised on okara, showed even more impressive results, with a viability of 12.98 log cfu/mL, a pH of 4.31, total acidity of 1.27%, and organic acid contents of 13.90 and 9.30 g/L, respectively. Over a 12-h fermentation period, cell viability values increased by 10.47-fold in free cells and 11.19-fold in synbiotics. Synbiotic supplementation of fermented soymilk proved more beneficial than free cells in terms of viability, acidity, and organic acid content. Furthermore, when synbiotic fermented soymilk was freeze-dried to simulate the digestive system in vitro, synbiotics and freeze-dried cells demonstrated superior gastrointestinal tract survival compared to free cells. Both the probiotic bacteria and the synbiotics exhibited cytotoxicity against colon and liver cancer cell lines, with half-maximal inhibitory concentrations ranging from 41.96 to 61.52 μL/well.

## Introduction

In recent years, consumer interest has surged in foods that go beyond simply fulfilling basic nutritional needs to also providing significant health benefits. These foods are valued for their potential to mitigate the risk of chronic diseases and enhance overall well-being by positively influencing metabolism, physiological functions, and overall health. Commonly known as functional foods, they represent a proactive approach to health maintenance and disease prevention by incorporating additional ingredients or enhancing existing ones to distribute specific health advantages^1^. Food is rich in bioactive compounds like vitamins C and E, carotenoids, flavonoids, phenolic acids, anthocyanins, and probiotics, all of which significantly contribute to health promotion. Responding to the rising consumer demand for these health benefits, the food industry is dedicated to developing products that effectively leverage these functional properties to enhance overall well-being^2^. As per the World Health Organization (WHO), probiotics are living microorganisms, and nowadays, the health benefits they offer are widely acknowledged. Probiotics can fight off harmful microbes, potentially lower the risk of cancer, enhance sugar metabolism, decrease cholesterol levels in the blood, bolster the immune system, and inhibit harmful pathogens. However, a major concern is ensuring the longevity of probiotics in food products during storage and processing. It's crucial that these bacteria reach the intestine alive and in sufficient numbers (between 10 6 and 10 9 log cfu/mL) to have positive effects on the consumer's health^3^.

Recently, probiotics have been playing an essential role in creating functional foods, dietary supplements, and even biopharmaceuticals. Many studies indicate that foods enriched with probiotics, like dairy and soybean products, are readily absorbed by the body, are full of nutrients, and offer various health benefits^4^. Supplementation with a combination of probiotics accelerates the development of gut microbiome maturation and reduces intestinal inflammation. Furthermore, through the fermentation of food, probiotics metabolize various bioactive substances, enhancing their contribution to human health^2^**.** Currently, probiotics are widely available to the public, as functional foods or dietary supplements have a fundamental role in both food science and clinical medicine. This underscores the imperative to advance the development of high-quality probiotics and associated products^5^.

Prebiotics are specialized types of fiber found in certain foods that nourish beneficial bacteria in the gut, promoting digestive health. They are essentially food for probiotics, helping them thrive and maintain a healthy balance in the gut microbiome. Prebiotics are compounds typically found in various plant-based foods such as whole grains, bananas, greens, onions, garlic, soybeans, and artichokes. In addition, probiotics and prebiotics are added to some foods and are available as dietary supplements^6^. In 2008, the Food and Agricultural Organization (FAO) of the United Nations (UN) held a Technical Meeting to revise the definition of prebiotics. The panel suggested redefining prebiotics as "a non-viable food component that confers a health benefit on the host associated with modulation of the microbiota”^7^. Prebiotic ingredients, such as inulin and fructooligosaccharides (FOS), may also improve the activity and survival of probiotic bacteria in fermented soymilk^8^. Moreover, the combination of prebiotics and probiotics results in a synbiotic effect on gut microbiota^9^.

Immobilisation serves as a beneficial method for protecting probiotics from bile salts, acids, and other elements in the gastrointestinal tract, thereby facilitating the delivery of higher concentrations of viable cells to the intestine. This process involves physical–chemical or mechanical techniques, where cells are enclosed within encapsulating materials possessing properties that minimize or prevent harm to the immobilized microorganisms^10^.

Soybeans (Glycine max) boast an abundance of nutrients like proteins, vitamins, and minerals, all of which can be extracted in water to produce soymilk. Additionally, soymilk stands as a favored option for individuals with lactose intolerance, milk protein allergies, or those following a vegetarian diet^11^. Soy products commonly undergo fermentation, a procedure that boosts the presence of bioactive compounds and enhances the sensory and phytochemical traits of the products. Typically, probiotic bacteria are employed in fermenting soy products^12^**.** Fermented soymilk stands out as a soybean-derived functional food recognized for its health advantages. The microorganisms employed in fermenting soymilk are pivotal for its final properties, as they enrich it with isoflavones, linked to various health benefits. These benefits include safeguarding against gastrointestinal disorders, cardiovascular diseases, and cancers^13^. Preparation of soymilk-based products with probiotics is reasonably a novel approach in the field of fermented functional foods^14^.

The current study aimed to assess natural material capacity as a probiotic cell immobiliser and to investigate the growth and metabolic features of probiotic bacteria during soymilk fermentation. As well as, to evaluate the survivability of immobilized cells in a simulated upper gastrointestinal environment.

## Materials and methods

### Probiotic bacterial strains and media used

Ten probiotic bacterial strains were used in the previous studies to ferment soymilk^15,16^. These strains were *Lactiplantibacillus plantarum* ATCC 14,917, *Lacticasibacillus casei* DS 20,011, *Lactobacillus acidophilus* ATCC 20,552, *Streptococcus thermophilus* DSM 20,259, and *Bifidobacterium longum* B 41,409) which collected from the Food Technology Research Institute, Agricultural Research Center in Giza, Egypt, while *Lactiplantibacillus plantarum* DSA 20,174, *Limosilactobacillus reuteri* NRRL B-14171, *Lactobacillus delbrueckii* subsp. *bulgaricus* DSMZ 20,080, *Lacticaseibacillus rhamnosus* NRRL B-442, and *Bifidobacterium bifidum* NRRL B-41410 were collected from the Dairy Department, National Research Center in Giza, Egypt. These strains have been used to ferment soymilk. de Man, Rogosa, and Sharpe (MRS) broth^17^ was used to maintain and preservative all probiotic bacterial strains except *Streptococcus thermophilus*. M17 agar medium^18^ was used for maintenance and preservative *Streptococcus thermophilus* strain. culture slants were maintained at 5ºC on a preservation media after incubation at 37°C for 48 h.

### Soymilk preparation

Soybean (*Glycine max*) seeds were collected from markets in Cairo, Egypt, and used to prepare soymilk. These seeds were washed and soaked overnight in distilled water at 5°C. After the segregation of water, the soybeans combined 1:5 (w/v) with distilled water. The resulting slurry was then filtered through a double-layer cheesecloth and sterilized at 121°C for 15 min^15^. The chemical composition of soymilk (%) had been analyzed in a previous investigation by Gad et al.^15^.

### Inoculum preparation

For bacterial standard inoculum, a single colony loop of each tested bacterial culture was inoculated into a 250 ml in volume Erlenmeyer flask containing 50 ml of MRS or M17 broth media. The inoculated flasks were incubated for 24 h at 37 °C under static conditions^19^. The contents of these flasks were used as standard inoculum (One milliliter contained 2.3 × 10^7^ colonies forming unit (cfu)/mL) for the fermentation process study.

### Soymilk fermentation process

The fermentation process was carried out in 250 ml plugged Erlenmeyer flasks, each containing 100 ml of soymilk and inoculated with 5% of the standard inoculum, and then incubated for 12 h at 37 °C^15,16^. Samples from each flask were taken every two hours for 12 h to determine cell growth, pH changes, and total acidity, as described below. The organic acid content was calculated using the methods described later.

### Agro-industrial byproducts and waste preparation

The agro-industrial byproducts and wastes (okara, whey protein, banana peels, apple pomaces, sugarcane bagasse, orange peels, and lemon peels) were used in this study. They were washed three times with distilled water before being dried in an oven (Agilent, USA) at 70°C until they reached a constant weight. The dried materials were finally crushed into powder with a kitchen mill (MEDIATECH (MT-CR40), Egypt)^20^. The powder was stored at -20°C and used for further investigation.

### Preparation of free and immobilized cells (synbiotic)

The techniques mentioned by Xiudong et al.^20^ were used to prepare both free and immobilized cells (synbiotic). In one hundred milliliters of MRS broth, probiotic bacterial strains were grown statically for 48 h at 37 °C. The cells were centrifuged for 15 min at 4 °C at 12,000 × g. The pellets were washed three times with sterile 0.85%(w/v) NaCl saline solution. An immobilization procedure was used, which involved autoclaving Erlenmeyer flasks with 100 ml of MRS broth at 121 °C for 15 min while adding 4% (w/v) agro-industrial powder for immobilization. Following this, aseptic transfer of 3% (v/v) activated cultures was made into the MRS broth containing agro-industrial powder, and the mixture was incubated for 48 h at 37 °C. The fermented medium was filtered through cheesecloth to collect the immobilization supports that were held on the cloth after the immobilization process was finished. To start the fermentation of soymilk, immobilized cells were washed three times with distilled water.

### Freeze drying of soymilk fermented

The soymilk fermented by immobilized bacteria on okara was transferred to − 80 °C for 18 h, and freeze-drying was followed on a BenchTop Pro (Virtis, SP Scientific, Warminster, PA, USA) freeze-dryer under vacuum (30–35 Pa) at condenser temperature − 10 °C, for 24 h. No cryoprotectants were used during freeze-drying^21^**.**

### Cell viable count (viability) and growth kinetics

The cell viability of probiotic bacterial cultures was determined using the pour plate method^22^ with MRS and M17 agar, which was incubated for 48 h at 37 °C. The cell viable count (viability) was calculated after 12 h of incubation at 37 °C^23^**.**

Plotting of the relationship between viability and time was done. Equations (1–3)^24^ were used to determine the specific growth rate (μG), doubling time (t_d_), and multiplication rate (MR) during the logarithmic phase.

1 $${\text{Specific}}\;{\text{growth}}\;{\text{rate}}\;\mu {\text{G}}\left( {{\text{h}}^{ - 1} } \right) = \left( {{\text{Ln}}\;{\text{x}}{-}{\text{Ln}}\;{\text{x}}0} \right)/\left( {{\text{t}} - {\text{t}}0} \right)$$

where: X = Amount of growth after t time (t) and X0 = Amount of growth at the beginning time (t0).

2 $${\text{Doubling}}\;{\text{time}}\;\left( {{\text{t}}_{{\text{d}}} } \right) = {\text{Ln}}\left( 2 \right)/\mu {\text{G}}$$

3 $${\text{Multiplication}}\;{\text{rate}}\left( {{\text{MR}}} \right) = 1/{\text{t}}_{{\text{d}}}$$

### Analytical methods

pH value and acidification kinetics: The pH of the fermented soymilk was measured with a calibrated digital pH meter (Model Adwa 800). The acidification kinetics of the yoghurt samples were calculated by evaluating their ΔpH is the difference in pH between inoculation and the stationary phase^25^, maximum acidification rate (V_max_), time (h) to reach maximum acidification rate (T_m_), and Time (h) to reach pH 4.6 (Te). The maximum acidification rate (V_max_) is the ratio of change in pH over time, expressed in absolute values (pH unit/min), and was calculated using the following Eq. 4 according to Bezerra et al.^26^**.**

4 $$\text{Vmax }=\text{ max}\frac{dpH}{dt}$$

Total acidity determination: The titratable acidity of the sample was measured using 10 ml of weighed sample into a conical flask, and three drops of phenolphthalein indicator were added and titrated with 0.1 ml of sodium hydroxide until a pink colour appeared. The titer value was recorded and was expressed as a percentage of lactic acid, acetic acid, and propionic acid^27^**.**

Organic acids productivity (g/L/h) was calculated as recommended by Abdel-Rahman et al.^28^ as the following Eq. 5.

5 $$\text{Organic acids productivity }(\text{g}/\text{L}/\text{ h})=\left(\frac{\text{Organic acids concentration }(\text{g}/\text{L})}{\text{Fermentation time }(\text{h}) }\right)$$

Chemical analysis of fermented soymilk: Total solids, fat, total nitrogen, and ash contents of the samples were determined according to the Association of Official Analytical Chemists^29^. The content of carbohydrates was calculated^29^ by subtracting the moisture, protein, fat, and ash content from the total mass. The energy value was calculated by using the formula^30^:

$${\text{Energy}}\;{\text{Value}} = \left( {{\text{Protein}} \times 4 + {\text{Fat}} \times 9 + {\text{Carbohydrate}} \times 4} \right)\,{\text{kcal}}/100_{{\text{g}}}$$

### Cytotoxicity evaluation

For cytotoxicity assay, the cells were seeded in a 96-well plate at a cell concentration of 1 × 10^4^ cells per well in 100 µl of growth medium. Fresh medium containing different concentrations of the test sample was added after 24 h of seeding. Serial two-fold dilutions of the tested compound were added to confluent cell monolayers dispensed into 96-well, flat-bottomed microtiter plates (Falcon, NJ, USA) using a multichannel pipette. The microtiter plates were incubated at 37 ºC in a humidified incubator with 5% CO_2_ for 24 h. Three wells were used for each concentration of the test sample. Control cells were incubated without a test sample. After incubation of the cells at 37 °C, for 24 h, the viable cells yield was determined by a colorimetric method^31^. After incubating for 24 h, the numbers of viable cells were determined by the (3-[4,5-dimethylthiazol-2-yl]-2,5 diphenyl tetrazolium bromide) (MTT) test. Briefly, the media was removed from the 96 well plate and replaced with 100 µl of fresh culture RPMI 1640 medium without phenol red then 10 µl of the 12 mM MTT stock solution (5 mg of MTT in 1 mL of PBS) to each well including the untreated controls. The 96 well plates were incubated at 37 °C and 5% CO_2_ for 4 h. An 85 µl aliquot of the media was removed from the wells, and 50 µl of DMSO was added to each well mixed thoroughly with the pipette, and incubated at 37 °C for 10 min. Then, the optical density was measured at 590 nm with the microplate reader (SunRise, TECAN, Inc, USA) to determine the number of viable cells, and the percentage of viability was calculated as [(ODt/ODc)] × 100% where ODt is the mean optical density of wells treated with the tested sample and ODc is the mean optical density of untreated cells. The relation between surviving cells and drug concentration is plotted to get the survival curve of each tumor cell line after treatment with the specified compound^32^**.**

The percentage of inhibition^33^ is presented in IC_50_ using linear regression y = ax + b,

Where y is 50% inhibition and x is sample concentration**.**

### Simulated oral digestion

Simulated saliva (290 mg/L α -amylase, 89.6 g /L KCl, 175.3 g/L NaCl, 88.8 g/L NaH_2_PO_4_, 20.0 g/L KSCN, 57.0 g/L Na_2_SO_4_, 84.7 g/L NaHCO_3_, 2.0 g/L Urea, pH 6.8). The 20 ml sample was mixed with 6 ml simulated saliva and placed in a flask with 40 ml distilled water. The sample was stirred and cultured for 5 min at 37℃ and taken out 10 ml. The reaction was terminated with 100 μl trifluoroacetate (TFA, 10% v/v) solution. The sample was centrifuged at 1000 g for 10 min. The supernatant was a simulated oral digestion sample^34^**.**

### Simulated gastric and intestinal stress tolerance tests

Acidic conditions were simulated by acidic MRS broth with pH adjusted to 3.5, 2.5, and 1.5 by adding 1 M HCl^35^**.** Simulated gastric juices were prepared fresh daily by suspending pepsin (Himedia-India) (3 g/L) in sterile saline and adjusting pH to 1.5 with 1 M HCl at 37 °C^36^.

### Simulated pancreatic juices

It was prepared fresh daily by suspending pancreatin USP (Himedia-India) (1 g/L) in sterile saline (0.5% NaCl w/v) with pH adjusted to 8.0 by adding 0.1 M NaOH at 37 °C^36^**.** A simulated bile salt solution was prepared by adding 0.1%, 0.2%, or 0.3% (w/v) bile salt (Himedia-India) to MRS broth. To test simulated gastric and intestinal stress resistance, 1 ml of fermented soymilk containing free cells, Immobilized, and freeze-dried (cell counts adjusted to approximately 9 log cfu/g) was incubated in the prepared acidic MRS broth, simulated gastric juices, pancreatic juices, and bile salt solution for 1 or 3 h at 37 °C. Survival was evaluated by plate count on MRS agar.

### Statistical analysis

Data were statistically evaluated using the IBM® SPSS® Statistics software version 20 on the premise of Duncan's Multiple Range Test at the 5% level^37^. All analyses were carried out in triplicate.

## Results and discussion

### Soymilk fermentation by free probiotic bacterial strains and immobilized cells (synbiotic)

The ten probiotic bacterial strains of *Lactiplantibacillus plantarum ATCC* 14,917 (S1), *Lactiplantibacillus plantarum* DSA 20,174 (S2), *Lacticaseibacillus casei* DSM 20,011 (S3), *Limosilactobacillus reuteri* NRRL B-14171 (S4), *Lactobacillus acidophilus ATCC 20,552* (S5), *lactobacillus delbrueckii sub sp. bulgaricus* DSMZ 20,080 (S6), *Lacticaseibacillus rhamnosus* NRRL B-442 (S7), *Streptococcus thermophilus* DSM 20,259 (S8), *Bifidobacterium bifidum* NRRL B-41410 (S9), and *Bifidobacterium longum* B41409 (S10) were used as free cells and immobilised probiotic bacterial strains (synbiotic) for soymilk fermentation (moisture, 93.16 ± 0.5; protein, 2.10 ± 0.24; fat, 2.13 ± 0.05; carbohydrates, 2.43 ± 0.34; ash, 0.18 ± 0.19; total solids, 6.84 ± 0.09 and energy, 37.29 ± 0.71 (kcal/100)^15^.

Results in Table 1 exhibited that the bacterial strains viability (ranged from 8.46 to 10.47 log cfu/mL) in fermented soymilk product (soy yoghurt) when soy milk inoculated with free cells and when treated with immobilized probiotic bacterial strains, the viability of the tested strains was ranged from 10.37 to 12.98 log cfu/mL. The highest viability was recorded in the presence of *Lacticaseibacillus rhamnosus* NRRL B-442 (S7) as a free cell (10.47 log cfu/mL) and/or an immobilized cell on okara (12.98 log cfu/mL). while the lowest viability was achieved by *Limosilactobacillus reuteri* NRRL B-14171 (S4) as a free cell (8.46 log cfu/mL) and* Lactobacillus acidophilus* ATCC 20,552 (S5) as an immobilized cell on a banana peel (10.37 log cfu/mL).

**Table 1:** Growth parameters values during soymilk fermentation by free, and immobilized cells.

Parameters	Treatments	Probiotic bacterial strains									
		S1	S2	S3	S4	S5	S6	S7	S8	S9	S10
Viability (log cfu/mL)	Free cells	9.49 ± 0.64	9.37 ± 0.72	8.77 ± 0.47	8.46 ± 0.77	8.79 ± 0.69	8.55 ± 0.52	10.47 ± 0.59	9.29 ± 0.72	9.39 ± 0.69	9.32 ± 0.72
	IC on OK	11.57 ± 0.73	11.48 ± 0.62	10.93 ± 0.52	10.72 ± 0.63	10.89 ± 0.71	10.73 ± 0.66	12.98 ± 0.47	11.48 ± 0.69	11.53 ± 0.76	11.57 ± 0.64
	IC on WP	11.54 ± 0.84	11.57 ± 0.87	10.63 ± 0.66	10.57 ± 0.82	10.53 ± 0.57	10.61 ± 0.72	11.72 ± 0.83	11.46 ± 0.88	11.36 ± 0.57	11.34 ± 0.82
	IC on BP	11.19 ± 0.59	11.16 ± 0.78	10.51 ± 0.72	10.57 ± 0.66	10.37 ± 0.84	10.63 ± 0.92	11.59 ± 0.72	11.21 ± 0.79	11.26 ± 0.81	11.28 ± 0.59
	IC on AP	11.29 ± 0.87	11.27 ± 0.59	10.77 ± 0.84	10.61 ± 0.83	10.73 ± 0.66	10.57 ± 0.88	11.57 ± 0.68	11.13 ± 0.84	11.26 ± 0.74	11.26 ± 0.63
	IC on SB	11.19 ± 0.57	11.21 ± 0.65	10.63 ± 0.73	10.59 ± 0.64	10.66 ± 0.68	10.57 ± 0.76	11.47 ± 0.71	11.17 ± 0.72	11.28 ± 0.63	11.26 ± 0.71
	IC on OP	11.14 ± 0.91	11.13 ± 0.92	10.77 ± 0.68	10.57 ± 0.72	10.66 ± 0.74	10.59 ± 0.84	11.29 ± 0.82	11.18 ± 0.66	11.12 ± 0.72	11.16 ± 0.58
	IC on LP	11.10 ± 0.82	11.12 ± 0.55	10.59 ± 0.82	10.53 ± 0.84	10.63 ± 0.62	10.53 ± 0.71	11.22 ± 0.92	11.17 ± 0.82	11.15 ± 0.66	11.11 ± 0.74
pH	Free cells	4.43 ± 0.53	4.41 ± 0.49	4.57 ± 0.61	4.55 ± 0.54	4.53 ± 0.39	4.59 ± 0.53	4.41 ± 0.56	4.45 ± 0.53	4.46 ± 0.71	4.49 ± 0.45
	IC on OK	4.39 ± 0.45	4.37 ± 0.53	4.41 ± 0.49	4.42 ± 0.62	4.40 ± 0.42	4.42 ± 0.43	4.31 ± 0.49	4.34 ± 0.62	4.37 ± 0.53	4.33 ± 0.57
	IC on WP	4.38 ± 0.61	4.39 ± 0.47	4.42 ± 0.42	4.40 ± 0.58	4.43 ± 0.38	4.45 ± 0.62	4.32 ± 0.39	4.37 ± 0.48	4.39 ± 0.61	4.35 ± 0.61
	IC on BP	4.39 ± 0.39	4.38 ± 0.54	4.43 ± 0.52	4.42 ± 0.47	4.44 ± 0.52	4.49 ± 0.39	4.32 ± 0.42	4.36 ± 0.55	4.37 ± 0.48	4.34 ± 0.39
	IC on AP	4.41 ± 0.42	4.40 ± 0.39	4.45 ± 0.57	4.40 ± 0.52	4.46 ± 0.64	4.48 ± 0.57	4.44 ± 0.38	4.47 ± 0.52	4.41 ± 0.66	4.45 ± 0.42
	IC on SB	4.43 ± 0.51	4.45 ± 0.51	4.48 ± 0.49	4.42 ± 0.63	4.46 ± 0.47	4.48 ± 0.49	4.44 ± 0.51	4.41 ± 0.61	4.46 ± 0.59	4.45 ± 0.62
	IC on OP	4.39 ± 0.37	4.41 ± 0.52	4.41 ± 0.53	4.41 ± 0.47	4.42 ± 0.52	4.40 ± 0.52	4.42 ± 0.62	4.41 ± 0.48	4.43 ± 0.47	4.41 ± 0.55
	IC on LP	4.40 ± 0.52	4.41 ± 0.57	4.41 ± 0.61	4.40 ± 0.53	4.43 ± 0.45	4.40 ± 0.51	4.42 ± 0.47	4.43 ± 0.39	4.41 ± 0.54	4.40 ± 0.47
Total acidity (%)	Free cells	0.99 ± 0.07	0.96 ± 0.09	0.84 ± 0.05	0.81 ± 0.07	0.84 ± 0.05	0.89 ± 0.05	1.12 ± 0.05	0.90 ± 0.05	0.96 ± 0.07	0.96 ± 0.07
	IC on OK	1.21 ± 0.07	1.19 ± 0.09	1.18 ± 0.09	1.12 ± 0.05	1.18 ± 0.07	1.18 ± 0.07	1.27 ± 0.07	1.19 ± 0.07	1.21 ± 0.08	1.21 ± 0.07
	IC on WP	1.18 ± 0.08	1.16 ± 0.07	1.00 ± 0.05	1.12 ± 0.07	1.18 ± 0.08	1.14 ± 0.08	1.18 ± 0.08	1.14 ± 0.07	1.14 ± 0.05	1.16 ± 0.07
	IC on BP	1.18 ± 0.07	1.16 ± 0.07	1.12 ± 0.07	1.10 ± 0.08	1.12 ± 0.07	1.10 ± 0.07	1.18 ± 0.07	1.16 ± 0.07	1.18 ± 0.07	1.16 ± 0.07
	IC on AP	1.14 ± 0.08	1.16 ± 0.08	0.99 ± 0.08	0.96 ± 0.08	0.99 ± 0.08	1.10 ± 0.08	1.16 ± 0.07	1.14 ± 0.07	1.12 ± 0.08	1.14 ± 0.08
	IC on SB	1.18 ± 0.07	1.18 ± 0.07	1.12 ± 0.07	1.16 ± 0.07	1.14 ± 0.07	1.14 ± 0.07	1.18 ± 0.07	1.16 ± 0.08	1.18 ± 0.08	1.14 ± 0.08
	IC on OP	1.14 ± 0.08	1.12 ± 0.08	0.99 ± 0.08	0.99 ± 0.08	0.99 ± 0.08	0.96 ± 0.08	1.16 ± 0.05	1.12 ± 0.08	1.14 ± 0.05	1.14 ± 0.07
	IC on LP	1.12 ± 0.08	1.12 ± 0.08	0.99 ± 0.08	0.96 ± 0.08	0.99 ± 0.08	0.93 ± 0.08	1.18 ± 0.05	1.16 ± 0.08	1.16 ± 0.05	1.14 ± 0.07

IC, Immobilized cells. OK, Okara; WP, Whey Protein; BP, Banana Peel; AP, Apple Pomace; SB, Sugarcane Bagasse; OP, Orange Peel; LP, Lemon Peel. *Lactiplantibacillus plantarum ATCC 14,917* (S1), *Lactiplantibacillus plantarum DSA 20,174* (S2), *Lacticaseibacillus casei DSM 20,011* (S3), *Limosilactobacillus reuteri NRRL B-14171 (*(S4), *Lactobacillus acidophilus ATCC 20,552* (S5),lactobacillus delbrueckii sub sp. bulgaricus DSMZ 20,080 (S6), *Lacticaseibacillus rhamnosus NRRL B-442* (S7), *Streptococcus thermophilus DSM 20,259* (S8), *Bifidobacterium bifidum* NRRL B-41410 (S9), and *Bifidobacterium longum B41409* (S10). The data is provided as mean ± SD.

The probiotic bacterial count should be viable and abundant (> 10^6^ cfu/mL) in the probiotic product at the time of consumption to exert beneficial health benefits^38^**.** Soymilk was considered to be a better substrate for the growth of the probiotic strains that grow more quickly in it than in cow’s milk^39^**.** Soymilk has been widely accepted as a probiotic carrier, it has been demonstrated that probiotic organisms are capable of utilizing sucrose, a major disaccharide found in soymilk^13^**.** The increased probiotic bacterial viability indicated that Lactobacilli and Pediococci were able to adapt and survive in soymilk. This is consistent with previous studies that have described soymilk as a good medium for Lactobacilli and Pediococci^40^**.** Furthermore, agro-industrial residuals such as lemons, oranges, and cereals contain high amounts of dietary fibers, sugars, minerals, and essential vitamins that facilitate the growth of probiotics^41^**.** It also has been reported that okara comprised 14.5–55.4% dietary fiber, 24.5–37.5% proteins, 9.3–22.3% lipids, and amounts of sugars, minerals, and essential vitamins Asuka^42^**.** These ingredients give the okara of potential prebiotic effect which benefits the growth of probiotics^43^.

The above results show that immobilised cells on okara (synbiotic) grow higher than free cells due to the availability of fibers, proteins, lipids, sugars, minerals, and essential vitamins in okara have been reported by^20^.

### Changes in pH, titratable acidity (TA), and organic acids content after soymilk fermentation by free and immobilized probiotics

Data in Table 1 showed changes in pH and titratable acidity (TA) of fermented soymilk by free cells and symbiotic (the immobilized probiotic bacterial strains) after 12 h of fermentation time. At the end of soymilk fermented with the free probiotic bacterial strains drastically dropped pH values ranging between 4.41 and 4.59 and the increase in TA values ranged from 0.81 to 1.12%. as well as, when used the immobilized probiotic bacterial strains, the pH values drastically dropped ranging between 4.31 and 4.49 and TA ranged from 0.93 to 1.27%.

The results showed a relationship between reduced pH and increased titratable acidity (TA) levels, along with a higher probiotic bacterial population, indicating that soymilk is suitable for supporting and sustaining probiotic bacteria^44^. Generally, a decrease in pH was accompanied by an increase in TA, reflecting the sample's acidity. This is due to the production of acids, primarily lactic acid, during fermentation. Additionally, the decrease in pH for all samples throughout the fermentation period might be attributed to the accumulation of lactic and organic acids produced by the probiotic bacteria, as well as their metabolic activity^12^.

**Figure 1:**
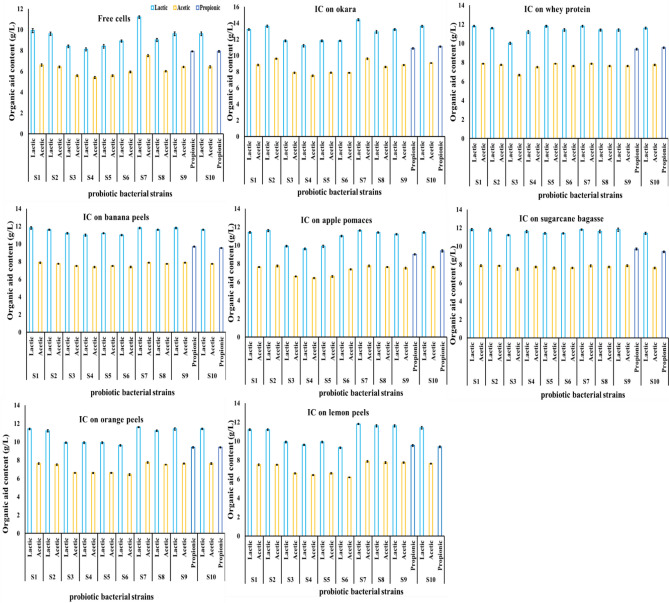
The change in organic acids content of fermented soymilk by free probiotic bacterial strains affected with fermentation time. *Lactiplantibacillus plantarum ATCC 14,917* (S1), *Lactiplantibacillus plantarum DSA 20,174* (S2), *Lacticaseibacillus caseiDSM 20,011* (S3), *Limosilactobacillus reuteri NRRL B-14171 (*(S4), *Lactobacillus acidophilus ATCC 20,552* (S5),lactobacillus delbrueckii sub sp. bulgaricus DSMZ 20,080 (S6), *Lacticaseibacillus rhamnosus NRRL B-442* (S7), *Streptococcus thermophilus DSM 20,259* (S8), *Bifidobacterium bifidum* NRRL B-41410 (S9), and *Bifidobacterium longum B41409* (S10).

**Figure 2:**
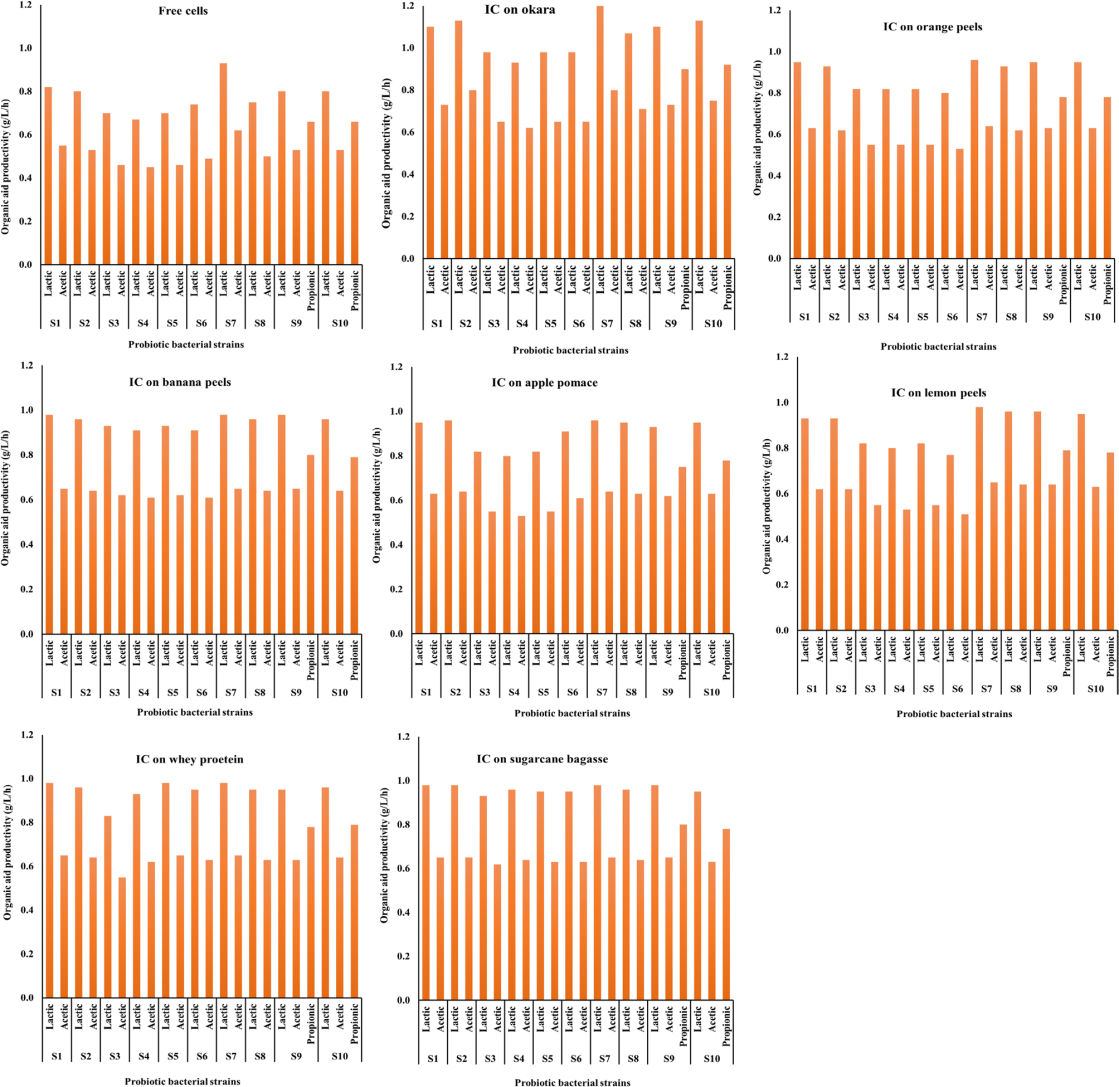
Organic acids (lactic and acetic or propionic acids) productivity during soymilk fermentation by free probiotic bacterial strains affected with fermentation time. *Lactiplantibacillus plantarum ATCC 14,917* (S1), *Lactiplantibacillus plantarum DSA 20,174* (S2), *Lacticaseibacillus caseiDSM 20,011* (S3), *Limosilactobacillus reuteri NRRL B-14171 (*(S4), *Lactobacillus acidophilus ATCC 20,552* (S5),lactobacillus delbrueckii sub sp. bulgaricus DSMZ 20,080 (S6), *Lacticaseibacillus rhamnosus NRRL B-442* (S7), *Streptococcus thermophilus DSM 20,259* (S8), *Bifidobacterium bifidum* NRRL B-41410 (S9), and *Bifidobacterium longum B41409* (S10).

Results in Figs. 1 and 2 demonstrated the formation of organic acids by all the tested free and synbiotic cells. The lactic acid concentration was higher than that of acetic acid after 12 h of fermentation. When fermented with free cells, the maximum concentrations of acetic and lactic acids were achieved by *Lacticaseibacillus rhamnosus NRRL B-442* (S7) at 7.50 and 11.20 g/L, with productivities of 0.62 and 0.93 g/L/h, respectively. The minimum concentrations of acetic and lactic acids were found in the sample fermented by *Limosilactobacillus reuteri NRRL B-14171 (*(S4), at 5.40 and 8.10 g/L, with productivities of 0.45 and 0.67 g/L/h. *Bifidobacterium bifidum* NRRL B-41410 (S9) and *Bifidobacterium longum B41409* were the only strains capable of producing propionic acid during fermentation, with a concentration of 7.91 g/L and a productivity of 0.66 g/L/h. In contrast, during soymilk fermentation for 12 h with immobilised probiotic bacterial strains, the lactic acid concentration was higher than that of acetic acid in all fermented samples. The maximum concentration of acetic and lactic acids and their productivity (Figs. 1 and 2) were achieved by *Lacticaseibacillus rhamnosus NRRL B-442* (S7), which was immobilised on okara (9.30 and 13.90 g/L) with productivity (0.77 and 1.15 g/L/h), while the minimum acetic and lactic acid concentration was (6.18 and 9.30 g/L) in the sample fermented by *Lactobaciilus delbrueckii subsp. bulgaricus* DSMZ 20,080 (S6), immobilised on lemon peels with productivity (0.51 and 0.77 g/L/h). *Bifidobacterium bifidum* NRRL B-41410 (S9) *and Bifidobacterium longum B41409 were* the only strains capable of producing propionic acid during fermentation, and their content ranged from 11.10 to 9.25 g/L for *Bifidobacterium longum B41409* (S10) immobilised on okara and Bifidobacterium bifidum NRRL B-41410 (S9) immobilised on apple pomaces, with a productivity of 0.92 and 0.75 g/L/h, respectively, for propionic acid.

The production of organic acids during fermentation is linked to a decrease in the pH, as was also observed in this study, and a very low pH value increases the concentration of organic acids in fermented soymilk, thereby enhancing the bactericidal effect of these acids^45^.

From the above results, it could be stated that the concentration of lactic acid was found to be higher than acetic acid in both cultures fermented by immobilised cells and free cells over 12 h, and the concentrations of lactic and acetic acids were higher in soymilk fermented by immobilised cells than free cells**.** During fermentation, a lower pH and higher concentrations of lactic and acetic acids and TA have been observed in soymilk inoculated with free probiotic bacterial cells compared with that inoculated with immobilised probiotic bacterial cells because of the higher growth rate and shorter lag phase of growth of immobilised cells than free cells. This finding might be mainly due to immobilised probiotic bacterial cells higher growth rate and higher substrate utilisation than free probiotic bacterial cells, leading to the increased production of organic acids (mainly lactic and acetic acids), which decreased the pH of soymilk^46,47^. The immobilised probiotic bacterial cells show significantly better growth compared with free probiotic cells, and that growth is accompanied by a higher production of lactic and acetic acids in soymilk, resulting in a lower final pH. soymilk inoculated with immobilised probiotic bacterial cells increased significantly faster than that of soymilk inoculated with free probiotic bacterial cells^41^. This could be due to the protection provided by the immobilisation matrix, which enhances cell survival and proliferation. Immobilised cells exhibit increased production of lactic and acetic acids in soymilk. This suggests that the immobilisation process may stimulate metabolic pathways responsible for acid production. Compared with fermentation with free cells, fermentation with immobilised cells shows higher fermentation rates, better substrate utilisation, lower cost, less product inhibition, a more favourable microenvironment for the cell, and other benefits^48^.

From the previous results, it could be concluded that *Lacticaseibacillus rhamnosus NRRL B-442* was the most effective probiotic bacterial strain when used for soymilk fermentation as a free cell and synbiotic (an immobilised cell on okara) for the next experiments.

### Soymilk fermentation by *Lacticaseibacillus rhamnosus NRRL B-442* as free cells and synbiotic, affected by fermentation time

The effective probiotic bacterial strain *Lacticaseibacillus rhamnosus NRRL B-442* was used as a free cell and an immobilised cell on okara (synbiotic) to ferment soymilk during interval times, and the parameters of viability, pH, TA, organic acid formation, and organic acid productivity were studied and tabulated in Table 2. Results point out that all determined parameters increased gradually during the fermentation period to reach their maximum value after 12 h, except pH values, which were gradually dropped. At the end of fermentation, the cell viability values were increased to 10.47 and 11.19-fold in free cells or synbiotic cells, respectively, and the TA values were increased from 0.09 to 1.12 and 1.27% in the presence of free cells or synbiotic cells, respectively. As well, the organic acid concentrations increased from 0.90 to 11.2, or 14.4 g/L, of lactic acid and 0.60 to 7.5, or 9.60 g/L, of acetic acid at the end of fermentation time in the presence of free cells or synbiotics, respectively. The pH values were dropped from 6.99 or 6.95 to 4.41 or 4.31 for free cells or synbiotics at the end of fermentation time, respectively.

**Table 2:** Growth parameters related to the most efficient probiotic bacterial stains used for the soymilk fermentation as free, and symbiotic (immobilized cells) during fermentation time.

Parameters	Treatments		Probiotic bacterial strains						
			0	2	4	6	8	10	12
Viability (log cfu/mL)	Free cells		1.00f. ± 0.37	2.13e ± 0.51	4.57d ± 0.53	7.71c ± 0.72	9.17b ± 0.85	10.26a ± 1.21	10.47a ± 1.19
	Synbiotic		1.16f. ± 0.41	2.21e ± 0.52	4.54d ± 0.61	8.25c ± 0.84	11.72b ± 1.14	12.91a ± 1.45	12.98a ± 1.47
pH	Free cells		6.99d ± 0.29	6.77 cd ± 0.64	6.41c ± 0.51	5.89bc ± 0.62	5.32b ± 0.71	4.57a ± 0.62	4.41a ± 0.56
	Synbiotic		6.95de ± 0.33	6.79d ± 0.66	6.31c ± 0.49	5.41b ± 0.57	4.59a ± 0.66	4.42a ± 0.65	4.31a ± 0.49
Total acidity (%)	Free cells		0.09f. ± 0.05	0.27e ± 0.04	0.54d ± 0.07	0.72c ± 0.05	0.96b ± 0.09	1.10a ± 0.07	1.12a ± 0.05
	Synbiotic		0.09f. ± 0.03	0.45e ± 0.09	0.66d ± 0.09	0.81c ± 0.05	0.99b ± 0.09	1.36a ± 0.09	1.27a ± 0.07
Organic acids content (g/L)	Free cells	Lactic acid	0.90f. ± 0.03	2.70e ± 0.13	5.40d ± 0.02	7.20c ± 0.07	9.60b ± 0.18	11.00a ± 0.11	11.2a ± 0.11
		Acetic acid	0.60f. ± 0.09	1.80e ± 0.09	3.60d ± 0.08	4.80c ± 0.09	6.42b ± 0.07	7.38a ± 0.09	7.50a ± 0.09
	Synbiotic	Lactic acid	0.90f. ± 0.03	4.50e ± 0.08	6.60d ± 0.11	8.10c ± 0.09	9.90b ± 0.16	13.90a ± 0.12	14.40a ± 0.13
		Acetic acid	0.60f. ± 0.08	3.00e ± 0.05	4.38d ± 0.09	5.40c ± 0.03	6.6b ± 0.09	9.30a ± 0.09	9.60a ± 0.10

Synbiotic, fermented soymilk by immobilized *Lacticaseibacillus rhamnosus NRRL B-442* on okara. The data is provided with mean ± SD. ^a,b^ The mean values within the same rows with different superscript letters are significantly different at *p* ≤ 0.05.

The growth parameters for the tested culture were calculated at the expansional phase of the growth curves, which are shown in Fig. S1. The specific growth rate (μG) was 0.196 and 0.21 h^-1^, the doubling time (t_d_) was 3.54 and 3.30 h, and the and the multiplication rate (MR) was 0.28 and 0.30 for soymilk fermented by *Lacticaseibacillus rhamnosus NRRL B-442* (S7) as a free and immobilised cell on okara, respectively. Specifically, Coda et al.^49^ demonstrated that probiotic bacterial strains were appropriate starters for non-dairy product fermentation. Probiotic bacterial fermentation is the simplest and safest way of preserving, and the addition of probiotics in yoghurt might produce dairy products with distinctive tastes, textures, and health advantages. Furthermore, de Mesquita et al.^23^ reported that probiotic bacterial strains cultivated in MRS medium had specific growth rates and doubling time values ranging between 0.12 and 0.21 h^−1^ and 1.38 and 2.44 h, respectively.

### Acidification kinetics related to soymilk fermentation

The acidification kinetics, including the maximum acidification rate (V_max_), time to reach Vmax (T_max_), and time to reach pH 4.5 (T_e_), were calculated for the fermented soymilk sample and presented in Table S1. The results showed that the soymilk fermented with both free cells and synbiotics exhibited an acidification rate (Vmax) of 0.007 pH/min. The time taken to reach the maximum acidification rate (T_max_) was 4 h for both free and synbiotic cells. In the case of soymilk fermented by *Lacticaseibacillus rhamnosus NRRL B-442* (S7), the time to reach pH 4.5 (T_e_) was 8 h for free cells and 7 h for synbiotic cells. The results agreed with Bezerra et al.^26^ revealed that demonstrated that the acidification kinetics of yoghurts from milk combinations exhibited Vmax values ranging from 15.1 to 18.9 unit pH/min, Tmax ranging from 179 to 210 min, and Te values ranging from 260 to 267 min. Similarly, Fawzi et al.^25^ reported that the highest acidification rate (V_max_) was 0.006 and 0.007 U/min, with a T_max_ of 4 h, and Te of 5 h and 4 h for samples fermented by *Lactiplantibacillus plantarum* and milk yoghurt cultures, respectively.

### Chemical and nutritional values of soymilk and fermented soymilk by free probiotics and synbiotics

The chemical composition and nutritional values were identified by several experiments done on soymilk and fermented soymilk. The data in Table 3 shows the chemical composition of fermented soymilk products produced by free cells and synbiotics. Fermented products were found to consist of varying percentages of protein, ash, fat, and carbohydrates as compared to non-fermented products. A decline in the levels of moisture, fat, and carbohydrates was noted, whereas there was an increase in the concentrations of total solids, protein, and ash in fermented soymilk. Total soluble solids content is an important parameter for beverage evaluation in the food industry. TSS in soymilk tells the lipid and protein contents of soymilk and also different others for nutritional value^50^**.** TSS is higher in the products that are always cherished by consumers. So, it was necessary to find them in the product. The TSS contents were 6.88, 8.43, and 8.58% for non-fermented soymilk and fermented soymilk by free cells and synbiotics, respectively. It is considered that the process of fermentation causes a breakdown of carbohydrate levels in soy milk, which could be the reason for the decreased TSS content in fermented soy milk^51^. The initial moisture content falls within the range of 93.12 to 91.57 and 91.422% for non-fermented soymilk, fermented soymilk by free cells, and synbiotics, and is similar to reports of Orhevba^52^**.** As a consequence of microbial cell proliferation, the decrease observed in moisture content as fermentation time progressed may be attributed to increased dry matter content^51^**.** In soy milk fermented for 12 h, a decrease in moisture content was also recorded. Desta et al.^53^ stated that the accumulation of nutrients was typically increased by a reduction in moisture. Moisture content decreased as fermentation time increased, while the overall solid content of soymilk fermentation increased. The results also revealed a development in the value of fermented soymilk products in protein and ash, with increased values reaching 1.87 and 1.88 folds for protein content and 4.05 and 4.68 folds for ash content as compared to non-fermented soymilk with fermented soymilk by free cells and synbiotic, respectively. The inorganic material left after the burning for the complete removal of water and organic matter in the food is known as ash. Soy milk is rich in calcium, iron, magnesium, and zinc, and all of these are important for the human body^54^. The ash content in non-fermented soy milk was 0.19%, while it was 0.77 and 0.89% in soymilk fermented by cells and synbiotics, respectively. The process of fermentation significantly increases the ash contents. The increase in ash content in fermented soy milk in comparison to non-fermented soy milk could be due to the reduction of certain other compounds, such as loss of moisture and breakdown of fat and carbohydrates^55^. The protein contents of a recent study were 2.12, 3.97, and 3.99% for non-fermented soymilk, fermented soymilk by free cells, and synbiotic. The estimation of total nitrogen content in food gives the estimation of protein in any food product, which is also considered a quality index. The protein concentration plays a vital role in determining the acid coagulation quality of protein gel products. Protein content was an important reflection of the nutritional value of fermented soymilk^56^. The process of fermentation affects the protein of soy milk by lactic acid bacteria converting protein into oligopeptides. The increase identified in fermenting soymilk protein content compared to soymilk could be attributed to certain anabolic processes leading to polymer buildup or microbial cell proliferation^57^.

**Table 3:** Chemical composition of soymilk, and fermented soymilk by free cells and synbiotic.

Treatments	Parameters (%)						
	Moisture	Protein	Fat	Carbohydrates	Ash	Total solids	Energy (K cal/100 g)
NFSM	93.12^a^ ± 2.14	2.12^b^ ± 0.21	2.17^a^ ± 0.19	2.40^a^ ± 0.14	0.19^b^ ± 0.17	6.88^b^ ± 0.24	37.61^b^ ± 0.71
Free cells	91.57^b^ ± 1.21	3.97^a^ ± 0.39	1.73^b^ ± 0.27	1.96^b^ ± 0.31	0.77^a^ ± 0.22	8.43^a^ ± 0.32	39.29^a^ ± 0.57
Synbiotic	91.42^b^ ± 1.09	3.99^a^ ± 0.51	1.79^b^ ± 0.17	1.91^b^ ± 0.24	0.89^a^ ± 0.19	8.58^a^ ± 0.19	39.71^a^ ± 0.62

NFSM, non-fermented soymilk. The data is provided with mean ± SD. ^a,b^ The mean values within the same columns or rows with different superscript letters are significantly different at *p* ≤ 0.05.

Soy milk is considered healthier as it is free from cholesterol and low in saturated fat. The fat contents of a recent study were 2.17, 1.73, and 1.79% for non-fermented soymilk and fermented soymilk by free cells and synbiotics, respectively. During the process of fermentation, the fat content improved slightly. This may be due to the increased lipolytic enzyme activity during fermentation that hydrolyzes fat components (triacylglycerol) into fatty acids and glycerol and is used as energy sources, as suggested by Astuti et al.^58^. During the fermentation process, probiotic bacterial strains can degrade fat or lipids into free fatty acids, so that the content of total fat is reduced and the content of free fatty acids is increased^59^. Also, the carbohydrate content in this study decreased significantly from 2.40 to 1.96 and 1.91% for non-fermented soymilk, fermented soymilk by free cells, and synbiotic at 12 h of fermentation, respectively. The decrease observed in the carbohydrate content of fermented soymilk as the fermentation period increased could be explained by the fermenting microorganisms activities, which utilised and transformed them into energy for growth and other cellular activities^60^. The energy values were 37.61, 39.29, and 39.71 kcal/100 g for non-fermented soymilk, fermented soymilk by free cells, and synbiotic. The higher energy value of the samples was a result of their higher protein and fat contents. The number of calories in a given food is termed the energy value of the food and is a good factor for comparing the true value of different foods3^30^**.**

### Effect of simulated gastrointestinal tract GI conditions on survivability of free, synbiotic, and encapsulated *Lacticaseibacillus rhamnosus NRRL B-442*

**Figure 3:**
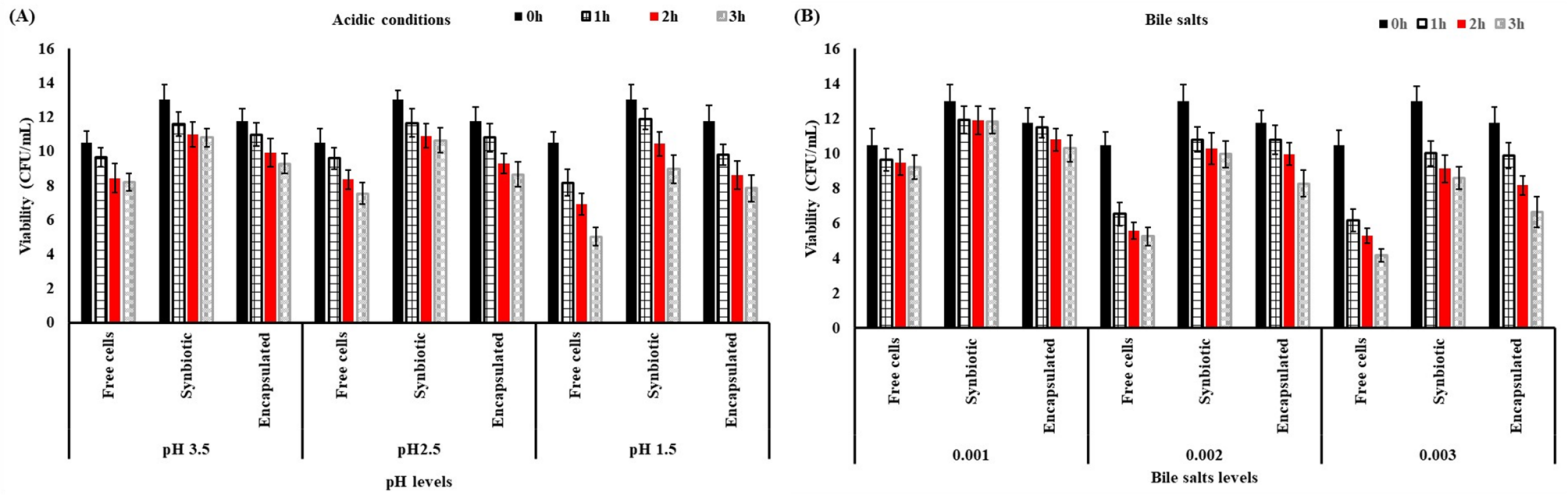
Effect of acidic condition (**A**) and bile salts (**B**) on survival of free cells, synbiotic, encapsulated *Lacticaseibacillus rhamnosus NRRL B-442*. FSMFC, fermented soymilk by free *Lacticaseibacillus rhamnosus NRRL B-442*.

The ability of probiotics to tolerate acidity is crucial, serving not only to withstand stomach conditions but also as a requirement in manufacturing acidic probiotic foods. The buffering capacity of the food, which is a major factor affecting pH, and the rate of gastric emptying may significantly influence cell survival in the GI tract^61^**.** The pH level of gastric juice stands as a pivotal factor in determining the viability of probiotic bacteria as they navigate from the stomach to the intestine. Results in Fig. 3A showed that the soymilk fermented by free, synbiotic, and encapsulated (freeze-dried) samples was put in an acidic solution at pH 1.5, 2.5, and 3.5 for 1, 2, and 3 h, respectively. The initial viability was 10.47, 12.98, and 11.73 log cfu/mL for soymilk fermented by free cells, synbiotic, and encapsulated cells, respectively. The results indicate that the viability of fermented soymilk by free *Lacticaseibacillus rhamnosus NRRL B-442* decreased from 10.47 to 9.62, 8.41, and 8.19 log cfu/ml under acidic conditions (pH 3.5), to 9.56, 8.34, and 7.51 log cfu/ml under acidic condition (pH 2.5), and 8.14, 6.89, and 4.98 log cfu/mL under acidic condition (pH 1.5), while the viability of fermented soymilk by synbiotic decreased from 12.98 to 11.57, 10.98, and 10.77 log cfu/mL under acidic condition (pH 3.5), to 11.63, 10.89, and 10.62 log cfu/mL under acidic condition (pH 2.5), and to 11.87, 10.41, and 8.93 log cfu/mL under acidic condition (pH 1.5), furthermore the viability of encapsulated cells decreased from 11.73 to 10.96, 9.89, and 9.26, and 10.77 log cfu/mL under acidic condition (pH 3.5), to 10.77, 9.26, and 8.63 log cfu/mL under acidic condition (pH 2.5), and 9.77, 8.57, and 7.82 log cfu/ml under acidic condition (pH 1.5) for 1, 2 and 3 h, respectively. These results are in agreement with Sidira et al.^62^ who reported that acidic conditions significantly reduce the number of both free cells and synbiotics. However, after 120 min at pH 2.0 and after 30, 60, 90, and 120 min at pH 1.5, the cell viability of synbiotics was significantly higher than that of free cells. Fijałkowski et al.^63^ found that the viability of *Lactobacillus* cells adsorbed on or entrapped in bacterial cellulose incubated in simulated gastric juices for 4 h is significantly higher than that of free cells, particularly for *Lactobacillus* cells entrapped in bacterial cellulose, which showed viability of more than 70% compared with less than 10% for free cells. Su et al.^64^ also found that probiotics can survive better in encapsulation than in free cells.

Bile salts, generated in the liver through cholesterol breakdown, act as surface-active agents. Hence, probiotics need to possess bile tolerance. The ability to hydrolyze bile salts is considered one of the key features of probiotic bacteria, according to the World Health Organisation, although not all probiotics have this ability (WHO and FAO, 2006)^42^**.** The mean intestinal bile concentration in the human gastrointestinal tract is estimated to be 0.3% w/v, although it may vary among individuals. Once the bacteria reach the intestinal tract, bile entering the duodenal section of the small intestine has been found to reduce the survival of bacteria. Therefore, this part of the study was conducted as an in vitro experiment to screen the three tested strains for their ability to tolerate bile concentrations of 0.3, 0.5, and 1.0% (w/v) to mimic approximate levels in the intestinal tract. The results obtained are plotted in Fig. 3B. The soymilk fermented by free cells, synbiotic cells, and encapsulated cells was treated with various concentrations of bile salt at 0.1, 0.2, and 0.3% for 1, 2, and 3 h, respectively. The initial viability was 10.47, 12.98, and 11.73 log cfu/ml for soymilk fermented by free cells, synbiotics, and encapsulated cells, respectively. The results indicate that the viability of fermented soymilk by free *Lacticaseibacillus rhamnosus NRRL B-442* decreased from 10.47 to 9.63, 9.47, and 9.18 log cfu/ml at 0.1% bile salts, to 6.51, 5.56, and 5.24 log cfu/mL at 0.2% bile salts, and 6.14, 5.28, and 4.16 log cfu/mL at 0.3% bile salts, while the viability of fermented soymilk by synbiotic decreased from 12.98 to 11.89, 11.87, and 11.82 log cfu/mL at 0.1% bile salts, to 10.77, 10.26, and 9.93 log cfu/mL at 0.2% bile salts, and 9.98, 9.11, and 8.56 log cfu/mL at 0.3% bile salts, furthermore the viability of encapsulated cells decreased from 11.73 to 11.47, 10.77, and 10.26 log cfu/mL at 0.1% bile salts, to 10.77, 9.93 and 8.26 log cfu/mL at 0.3% bile salts, and to 9.87, 8.16, and 6.63 log cfu/mL at 0.3% bile salts, for 1, 2, and 3 h, respectively. The results showed that 0.1% bile salt exerted no significant effect on the survival of free cells or synbiotics in soymilk. When the concentration of bile salt increased to 0.2% and 0.3%, the survival of free cells or synbiotics in soymilk significantly decreased. However, synbiotics showed a significantly higher number of viable cells compared with free cells. These results were in line with the observations of Sidira et al.^62^ who reported that the viable cell count of *L. case* ATCC399 immobilised in apple pieces decreased from 9.30 log cfu/mL to 6.23 log cfu/mL after 4 h of incubation in 1% bile salt solution, whereas the viable cell count of free L. *. case* ATCC399 decreased from 9.16 log cfu/mL to 3.66 log cfu/mL. *L. plantarum* NCIMB 8826 immobilised within malt and barley cereal fibre could improve its viability in a bile salt solution. The research findings suggest that okara holds potential as an innovative immobilisation carrier, amplifying the growth and glucosidic isoflavone bioconversion capabilities of *L. plantarum* 70,810 in soymilk. Furthermore, it aids in sustaining cell viability during simulated gastric and intestinal conditions^20^.

According to these results, we postulated that synbiotics showed improved tolerance to bile salt stress compared with synbiotics, which might be due to the physical entrapment of bile salts into the dietary fibre of okara.

### Free, synbiotic, and encapsulated *Lacticaseibacillus rhamnosus NRRL B-442* Survival during in vitro digestion

**Figure 4:**
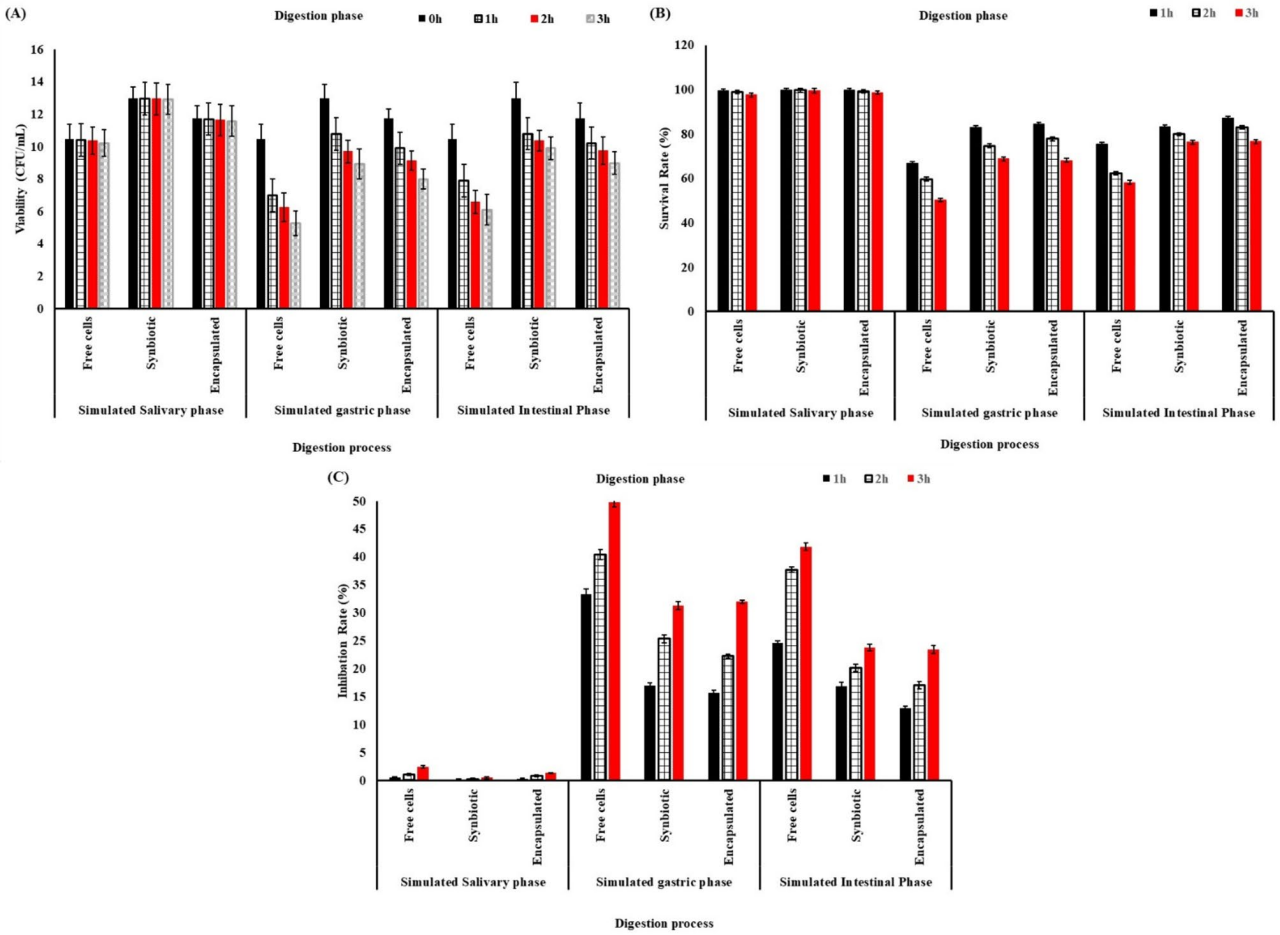
In Vitro Survival of free *Lacticaseibacillus rhamnosus NRRL B-442* cells, synbiotic and encapsulated under digestion process conditions (salivary, gastric and intestinal phases). (**A**) Viability (log cfu/mL), (**B**) Percentage survival rate and (**C**) Percentage of inhibition rate.

To investigate the potential protective effect of synbiotic and freeze-drying on the viability of *Lacticaseibacillus rhamnosus NRRL B-442* cells during GI transit, both free cells and synbiotic and encapsulated cells were subjected to a static in vitro digestion model. For comparison purposes, free cells, symbiotic cells, and encapsulated cells were also tested in the same digestion process (salivary, gastric, and intestinal phases), as illustrated in Fig. 4A–C.

The simulated salivary phase had no significant effect on the viable cell counts of fermented soymilk by free, synbiotic, or encapsulated cells. The viability of fermented soymilk by free *Lacticaseibacillus rhamnosus NRRL B-442* decreased from 10.47 to 10.41, 10.36, and 10.21 log cfu/mL, with a survival rate of 99.42, 98.94, and 97.51%, while the viability of fermented soymilk by a synbiotic decreased from 12.98 to 12.96, 12.94, and 12.91 log cfu/mL, with a survival rate of 99.84, 99.69, and 99.46%; furthermore, the viability of encapsulated cells decreased from 11.73 to 11.69, 11.63, and 11.57 log cfu/mL, with a survival rate of 99.66, 99.14, and 98.63% for 1, 2, and 3 h, respectively (Fig. 4A,B)**.** On the other hand, the inhibition rate of viable cell count during the salivary phase was 0.58, 1.06, and 2.49% for fermented soymilk by free *Lacticaseibacillus rhamnosus NRRL B-442*, 0.16, 0.31, and 0.54% for fermented soymilk by synbiotic, and 0.34, 0.86, and 1.37% for encapsulated cells for 1, 2, and 3 h, respectively (Fig. 4C)**.** The results agreed with Nelios et al.^65^ who reported that incubation in the simulated salivary phase had a non-significant effect on the viable cell counts of free, synbiotic, and encapsulated cells.

Through the *in* vitro gastric phase, the viable cell counts of fermented soymilk by free, synbiotic, and encapsulated cells decrease significantly during the *in* vitro gastric phase (Fig. 4A–C). The viability of fermented soymilk by free *Lacticaseibacillus rhamnosus NRRL B-442* decreased from 10.47 to 6.98, 6.24, and 5.26 log cfu/mL, with a survival rate of 66.66, 59.6, and 50.23%; the viability of synbiotic decreased from 12.98 to 10.77, 9.69, and 8.92 log cfu/mL, with a survival rate of 82.97, 74.65, and 68.72%; and the viability of encapsulated cells decreased from 11.73 to 9.89, 9.12, and 7.98 log cfu/mL, with a survival rate of 84.31, 77.74, and 68.03% for 1, 2, and 3 h, respectively (Fig. 4A,B)**.** On the other hand, the inhibition rate of viable cell count during the gastric phase was 33.34, 40.4, and 49.77% for fermented soymilk by free *Lacticaseibacillus rhamnosus NRRL B-442*, 17.03, 25.35, and 31.28% for synbiotic, and 15.69, 22.26, and 31.97% for encapsulated cells for 1, 2, and 3 h, respectively (Fig. 4C). The tolerance to acidity is essential for probiotics, as it plays a vital role in both withstanding gastric stress and aiding in the manufacturing of acidic probiotic food products. Furthermore, the results follow the results of Shi et al.^66^ who demonstrated that the use of polymers for the encapsulation of probiotics protects and maintains the desired viability of probiotics in an acidic environment, The encapsulation of the cells with hydrogel materials improves their viability and stability in a low-pH medium. The survival rate of probiotics in the case of sodium alginate was higher as compared to carrageenan. There was a low survival rate when considering cells that were not encapsulated. The results demonstrated that encapsulation protects probiotics in simulated gastric conditions. Afzaal et al.^67^ reported poor survival with free probiotics. The results also found that in simulated gastric conditions, probiotics survive better when encapsulated with different materials^68^.

In the Vitro intestinal phase, the viable cell count of fermented soymilk by free cells, synbiotics, and freeze-dried fermented soymilk decreased significantly during the In Vitro intestinal phase. The viability of fermented soymilk by free *Lacticaseibacillus rhamnosus NRRL B-442* decreased from 10.47 to 7.89, 6.57, and 6.09 log cfu/mL, with survival rates of 75.35, 62.27, and 58.16%; by synbiotic, they decreased from 12.98 to 10.79, 10.36, and 9.89 log cfu/mL, with survival rates of 83.13, 79.81, and 76.19%; and the viability of encapsulated cells decreased from 11.73 to 10.21, 9.73, and 8.98 log cfu/mL, with survival rates of 87.04, 82.94, and 76.55% for 1, 2, and 3 h, respectively (Fig. 4a,b). While the inhibition rate of viable cell count during the salivary phase was 24.65, 37.73, and 41.84% for fermented soymilk by free *Lacticaseibacillus rhamnosus NRRL B-442*, 16.87, 20.19, and 23.81% for synbiotic, and 12.96, 17.03, and 23.45% for encapsulated cells for 1, 2, and 3 h, respectively (Fig. 4c).

The cell viable count in soymilk by synbiotic and encapsulated cells was significantly higher at both time points compared with that in soymilk by free *Lacticaseibacillus rhamnosus NRRL B-442*. This result differs from those reported Sidira et al.^62^ who found that simulated pancreatic juice exerts no effect on the survival of immobilised *L. case* ATCC399 but significantly affects the viability of free *L*. *case* ATCC399. The observed result could be due to variations in strains or substrate materials utilized. Maintaining cell viability under gastric and intestinal conditions is crucial for achieving the intended advantages of probiotics. The composition of the intestinal solution significantly impacts the liberation of encapsulated cells. Encapsulating polymers in a high-pH solution start to dissolve, which causes a rapid release of cells from the beads in the intestinal sector^69^.

### Anticancer activity of fermented soymilk

Cancer, characterised by uncontrolled cell growth, leads to organ deterioration and eventual mortality. The mortality rate due to different cancer types continues to increase annually. Current chemotherapy and radiation therapy for cancer patients harm both tumour and normal cells^70,71^ Current research endeavours to develop anti-tumour compounds with reduced side effects compared to existing synthetic drugs. In this experiment, the MTT assay was used to determine the inhibitory effect of soymilk extracts fermented by free cells, prebiotics (okara), and synbiotics against colon cancer cells (HT-116) and liver cancer cells (HepG2) cell lines. Both HT‐116 cells and HepG2 cells were exposed to various concentrations of soymilk extracts fermented by free, prebiotic, and synbiotic means ranging from 0.2 to 100 µl/10^4^ cells, and cell viability was assayed by the amounts of neutral red uptake in the cells shown in Fig. S2A,B. Results indicated that the viability of colon and liver cancer cell lines of HT‐116 cells and HepG2 cells was completely grown after exposure up to 0.78 µl /10^4^ cells of soymilk extracts fermented by free cells and synbiotic against colon cancer cell (HT-116) cells, while at high concentrations (100 µl /10^4^ cells) of soymilk extracts fermented by free cells and synbiotic, it was observed that the viability of HT‐116 cells was inhibited with 78.49 and 81.89%, respectively. On the other hand, cells were completely grown after being exposed to up to 3.12 and 1.56 µl /10^4^ cells of soymilk extract fermented by free cells and synbiotic against liver cancer cell (HepG2) cells. While at high concentrations (100 µl/10^4^ cells) soymilk extracts fermented by free cells and symbiotic, it was observed that the viability of HT‐116 cells was inhibited at 72.81 and 75.46%, respectively. The inhibitory mechanisms of *Lactobacillus* against colorectal cancer cells include reducing tumor-promoting enzymatic activity, binding to mutagens, Computed death's half-maximal (50%) inhibitory concentration (IC_50_) was performed using Excel (Fig. S2A, B). Results showed that the IC_50_ values of soymilk extracts fermented by free cells and synbiotics against HCT-116 were 47.02 and 41.96 µl/well, respectively (Fig. S2A). Whereas, the IC_50_ values were 61.52 and 58.89 (µl/well) against HepG2 treated by soymilk extracts fermented by free cells and synbiotics (Fig. S2B). A high determination coefficient (R^2^) ranged from 0.93 to 0.94 for HCT-116 and from 0.92 to 0.93 for HepG2, which were treated by soymilk extracts fermented by free cells and symbiotic, respectively (Fig. S2 A, B).

These results are in agreement with Khan and Kang^72^ who revealed that soymilk fermented with probiotic bacterial strains showed antiproliferative effects on human colon cancer (HCT-116). Zhang et al.^73^ found that soymilk fermented with probiotic bacterial strains showed an antiproliferative effect on human liver cancer (HepG2). These results might be due to the increase in the aglycone isoflavones, which Genistein also inhibited the cell migration of HepG2 cells, as evident from the wound healing assays**.** Probiotics are thought to be the most important microbes for anticarcinogenic activity^74^**.** By altering the gut's surrounding conditions and lowering the population or metabolic activity of bacteria competent to create carcinogenic chemicals. Le et al.^75^ compared the effects of soymilk fermented by *L. rhamnosus* GG and fermented soymilk fortified with xylooligosaccharides (XOS) on the growth of colon cancer cells. The fermented soymilk increased dextran, folate, and aglycone levels and reduced the growth of the HCT116 cell lines.

**Figure 5:**
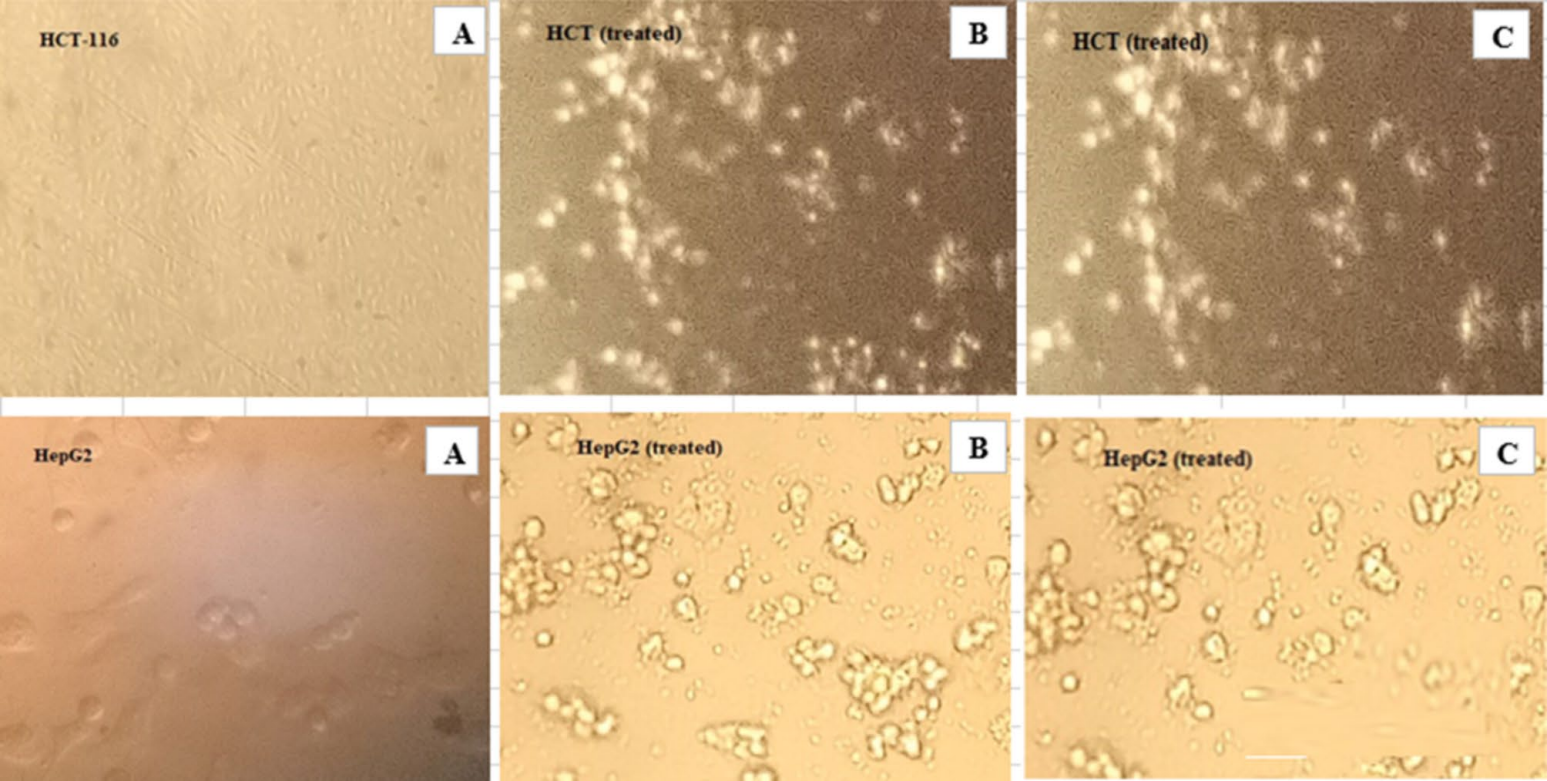
Representative photomicrograph of HCT-116, and HepG2 cells morphology before treated (**A**), and after treated by soymilk extracts fermented by free (**B**), and symbiotic (**C**), photographed with an inverted phase-contrast microscope (magnification 100 ×).

Images obtained by microscopy (Fig. 5A) depict the morphological alterations seen in HCT-116 and HepG2 before being treated, while images in (Fig. 5B,C) show morphological alterations seen in HCT-116 before and after being treated by soymilk extracts fermented by free cells and symbiotic, respectively. In addition, Kim et al.^76^ mentioned the role of probiotic bacteria in suppressing carcinoma cell growth, strengthening the immune system, repressing pro-carcinogenic enzyme activity, removing mutational molecules, and inducing the secretion of cytokines such as TNF-α (Tumor Necrosis Factor).

## Conclusion

The findings of the study indicated that soymilk fermented by probiotic bacterial strains as immobilised cells showed an increase in total acidity, higher productions of lactic and acetic acids, and a faster decrease in pH compared with using probiotic bacterial strains as free cells. In addition, agro-industrial residuals were used as carriers for probiotic bacterial strains such as Okara, and whey protein is a food-grade-quality, cheap, and abundant cell support for probiotic bacterial strain immobilization. Cells are firmly and easily immobilised onto agro-industrial residuals because of their vacuous and porous structure. Synbiotic showed a faster growth rate and a shorter lag phase of growth during soymilk fermentation. The immobilisation method was found to be very effective in increasing the viability of probiotic bacteria compared with free cells. The data from the viability study showed that the immobilisation technique provided high probiotic protection, as the number of live microorganisms was at a high level after 3 h of probiotic exposure in a simulated small intestinal environment. This indicates that the capsules successfully transported the live microorganisms through the acidic gastric environment to the small intestine, where they can manifest their beneficial effects on the human body. In addition, fermented soymilk by free cells and synbiotics showed anticancer properties against colon cancer cells (HT-116) and liver cancer cells (HepG2).

### Supplementary Information

Supplementary Information.

## Data Availability

The datasets used and/or analyzed during the current study are available from the corresponding author upon reasonable request.
