# Supplementary material for: In vitro digestive system simulation and anticancer activity of soymilk fermented by probiotics and synbiotics immobilised on agro-industrial residues
Source: Sci Rep. 2024 Aug 9;14:18518. doi: 10.1038/s41598-024-68086-3 (PMC11316043; doi:10.1038/s41598-024-68086-3)
Supplement: Supplementary file 1 — Supplementary Information. [file 41598_2024_68086_MOESM1_ESM.docx]

**Table S1.** Parameters of the kinetics of acidification during soymilk fermentation by free cells and synbiotic.

| **Treatments** | **Kinetics of acidification** | | | |
| --- | --- | --- | --- | --- |
|  | **ΔpH** | **V_max_ (dpH/dt)** | **T_max_** | **T_e_ (pH 4.5)** |
| Free cells | 0.26 | 0.007 | 4 | 8 |
| Synbiotic | 0.24 | 0.007 | 4 | 7 |

ΔpH, difference in pH between inoculation and the stationary phase; V_max_, Maximum acidification rate; T_m_, Time (h) to reach maximum acidification rate; T_e_, Time (h) to reach pH 4.5.

**
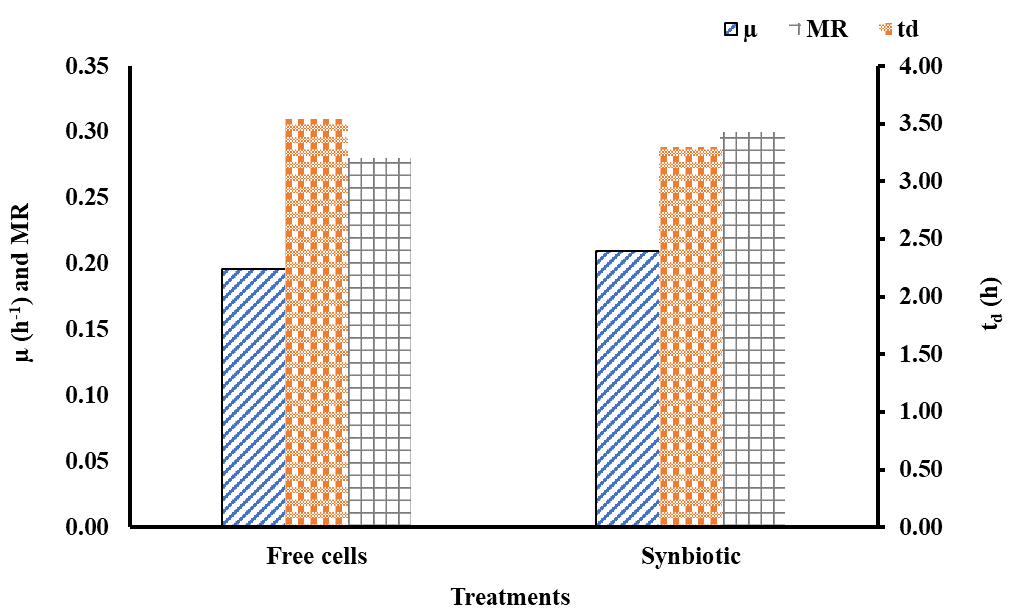
**

**Figure S1** Growth kinetics (μG, t_d_, and MR) of free probiotic strain were calculated during the log phase of soymilk fermentation.

μG, Specific growth rate; t_d,_ doubling time; MR, multiplication rate.


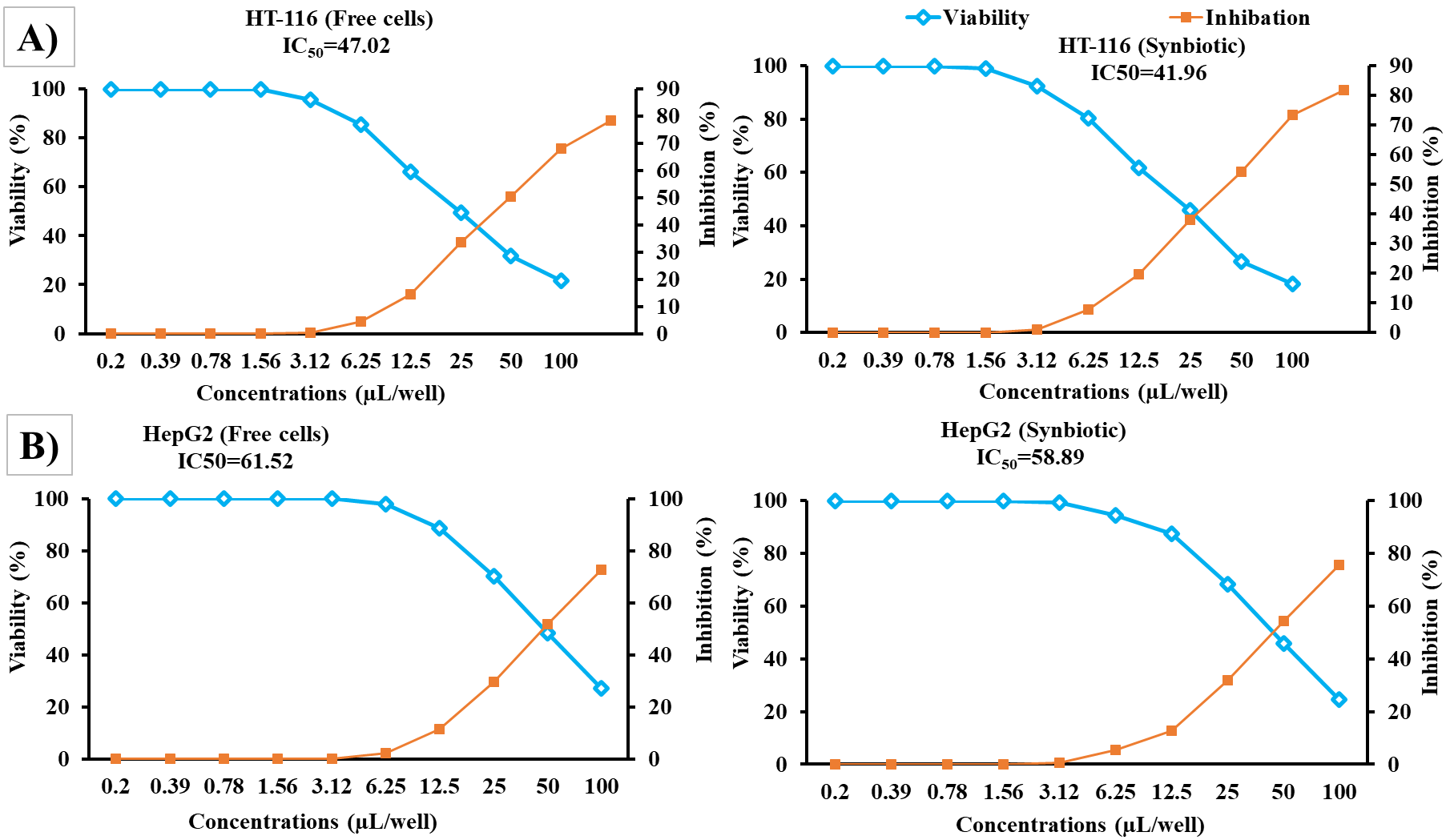


**Figure S2** Colon cancer cell HT-116 (A) and liver cancer cell HepG2 (B) cell lines viability (%) and half-maximal inhibitory concentration (IC_50_) after treatment with varying concentrations (0.2–100 μL/well) of soymilk fermented with free cells, and symbiotic.
